# Recombinant human IGF-1 alleviates dyslipidemia induced by lactational maternal dietary restriction

**DOI:** 10.3389/fped.2026.1829936

**Published:** 2026-06-04

**Authors:** Xin Liu, Yuanyuan Ma, Hong Cui, Qi Feng

**Affiliations:** 1 Department of Pediatrics, Beijing Friendship Hospital, Capital Medical University, Beijing, China; 2 Neonatal Intensive Care Unit, Department of Pediatrics, Peking University First Hospital, Beijing, China; 3 Laboratory of Animal Facility, Peking University First Hospital, Beijing, China

**Keywords:** extrauterine growth restriction, IGF-1, lactational dietary restriction, lipid metabolism, rat animal model

## Abstract

**Background:**

Extrauterine growth restriction (EUGR) critically impacts preterm infants, often due to nutritional deficiency. Lactational protein restriction can disrupt offspring glucose/lipid metabolism by altering gene expression and increasing branched-chain amino acid catabolism.

**Objective:**

This study aimed to investigate the effects of postpartum maternal dietary restriction (MDR) on growth and lipid metabolism in Sprague-Dawley (SD) rat offspring, and to explore the underlying mechanisms through Agilent microarray-based transcriptomic analysis.

**Methods:**

An EUGR rat model was established by maternal dietary restriction. Body weight, body length, serum insulin-like growth factor-1 (IGF-1), and lipid profiles were monitored from postnatal day 1 to day 21. To evaluate therapeutic effects, a separate cohort of EUGR pups received recombinant human IGF-1 (rhIGF-1) or PBS. Liver transcriptomic analysis was conducted to identify differentially expressed genes. Based on transcriptomic results, lipoprotein lipase (*LPL*) was overexpressed in HepG2 cells for *in vitro* functional validation.

**Results:**

At 3 weeks of age, EUGR pups showed significantly lower body weight and length than controls (*p* < 0.01). Serum triglycerides (TG) were reduced in EUGR rats (*p* < 0.05), whereas total cholesterol and HDL-C were elevated. rhIGF-1 treatment did not improve growth, but significantly reduced HDL-C compared with PBS. Liver transcriptomics revealed altered expression of lipid-metabolism genes in EUGR animals; notably, *LPL* expression was increased. In HepG2 cells, *LPL* overexpression significantly downregulated the lipogenic genes *ACACA* and *SCD* (*p* < 0.01).

**Conclusion:**

Maternal dietary restriction induces EUGR in rats, characterized by growth impairment and dyslipidemia. rhIGF-1 treatment failed to improve growth but modulated HDL-C levels. Liver transcriptomics in EUGR animals show upregulation of *LPL*, and *in vitro* experiments demonstrate that *LPL* can downregulate *ACACA* and *SCD*, suggesting that the *LPL*/*ACACA*/*SCD* pathway may underlie the observed triglyceride alterations in EUGR.

## 1 Introduction

Extrauterine Growth Restriction (EUGR) refers to early postnatal nutritional insufficiency in preterm infants, resulting in body weight below the 10th percentile (or Z-score ≤ −1.28) at discharge or at 36 weeks corrected gestational age (1–3). Its incidence remains high: approximately 91% of very preterm infants in NICUs develop EUGR by 36 weeks (4), and a multicenter study reported 86.2% (5). EUGR impacts not only physical growth and neurological development in childhood (6) but also leads to long-term adverse outcomes such as hyperglycemia, hypertension, and elevated inflammation in adolescence (7). Childhood blood glucose and lipid levels are associated with neonatal EUGR (8). Both excessive and restricted perinatal growth can affect adipose tissue synthesis, increasing the risk of adult metabolic syndrome (9, 10). Infant weight and metabolic status in early life are closely linked to long-term metabolic outcomes (11, 12).

Both prenatal and early postnatal nutrition can induce changes in adipose tissue and metabolic programming (13, 14). Clinical studies show lower total cholesterol (TCHO) and high-density lipoprotein (HDL) in cord blood of IUGR preterm infants (15), and term newborns with postnatal growth restriction exhibit reduced fat mass percentage and triglyceride content (16), correlating with poor growth (17–19). However, the mechanisms of these lipid disorders remain unclear. Animal models of maternal dietary restriction during lactation result in offspring growth restriction, reduced body weight, decreased muscle mass, and behavioral changes (20). Studies by Lee et al. (21) and Eleftherios et al. (22) report abnormal offspring blood lipid indices from dietary restriction during pregnancy or lactation; lactation-only restriction causes more severe reductions. Maternal protein restriction during lactation alters expression of glucose/lipid metabolism genes, promotes branched-chain amino acid catabolism, and modifies hepatic lipid phenotypes (23, 24). Therefore, early-life interventions targeting weight and lipid metabolism may be a critical window for improving adult metabolic disorders.

The growth restriction induced by maternal dietary restriction during lactation in rats mimics the postnatal nutrient deficiencies leading to growth restriction in preterm infants. Research using this model closely approximates clinical reality. The liver, as the central organ for metabolism, integrates signals, regulates glucose and triglyceride production, and stores fuel, making it key for studying glucose and lipid metabolism (25).

Insulin-like Growth Factor 1 (IGF-1) is a 70-amino-acid mitogenic hormone primarily produced by the liver (26). It mediates growth hormone effects, regulating biological growth and participating in glucose and lipid metabolism (27, 28). IGF-1 is a key endocrine determinant of fetal growth (29), serving as the primary growth hormone during gestation and increasing notably in mid-to-late pregnancy to accelerate fetal growth (30). It also promotes postnatal growth; poor growth in very preterm infants, despite improved early nutrition, may be related to low IGF-1 levels. Early postnatal growth velocity and weight gain correlate positively with serum IGF-1 (29, 31). Exogenous IGF-1 supplementation increases food utilization efficiency and weight gain in rats (32).

Based on previous research, EUGR is a critical issue in preterm infants, primarily caused by early postnatal nutritional deficiency. IGF-1, a key growth hormone during fetal and neonatal periods, plays an irreplaceable role in early growth and metabolism. This study hypothesizes that maternal dietary restriction during lactation induces growth restriction and lipid metabolism disorders in offspring, possibly via altered hepatic lipid metabolism genes. Exogenous IGF-1 supplementation may be a potential therapeutic strategy.

## 2 Materials and methods

### 2.1 Experimental animals

The offspring pups used in this study were obtained from pregnancies of Sprague-Dawley (SD) rats housed and bred in the Laboratory Animal Center of Peking University First Hospital. Specific pathogen-free (SPF) grade pregnant female SD rats were provided by the Laboratory Animal Center of Peking University First Hospital and purchased from Beijing Vital River Laboratory Animal Technology Co., Ltd. (License number SCXK (Jing) 2016-0011).

### 2.2 EUGR model construction

Pregnant rats were acclimated for one week pre-experiment under standard conditions: 20–22 °C, 65%–70% humidity, 12-hour light/dark cycle, with *ad libitum* access to food and water during gestation. On postnatal day 1 (PND1), litters were adjusted to 10 similar-weight pups per dam; excess pups were euthanized. Dams were matched by postpartum body weight and divided into two groups: Control group (Ctrl group, *n* = 3 litters, with 3 pups per litter): free access to diet throughout lactation; EUGR group (*n* = 3 litters, with 3 pups per litter): restricted to 50% of the matched control's daily intake throughout lactation. All subsequent metabolic and phenotypic analyses in this study were performed exclusively on male offspring. This design was implemented to control for the significant confounding effects of cyclical female sex hormones on lipid metabolism and to align with established models in developmental programming research (33, 34). To this end, on PND22, all offspring were euthanized. Male offspring (*n* = 9 from Ctrl group dams, *n* = 9 from EUGR group dams, randomly selected) were euthanized under deep anesthesia induced by 4% isoflurane inhalation, followed by tissue harvest via cardiac perfusion for downstream analyses (serum, liver, etc.). Female offspring were not included in the analytical cohorts; they were humanely euthanized using a CO2 displacement method at a filling rate of 50% of the chamber volume per minute. All procedures involving animals were conducted in compliance with relevant regulations and guidelines and were approved by the Peking University First Hospital Animal Ethics Committee (Approval No. J202152).

#### 2.2.1 Physical growth measurement

From postnatal day 1 to day 21, the body length and body weight of pups in both groups were measured every other day. On lactation day 22, male offspring were anesthetized with 4% isoflurane. After confirmation of a state of deep anesthesia, cardiac perfusion was performed immediately. Major organs (including liver, brain, heart, kidneys, hindlimb muscles, and tibia) were immediately dissected and weighed.

#### 2.2.2 Serum IGF-1 Level measurement

Blood samples were collected from male pups via the submandibular venous plexus. For each collection, 50 μL of blood was drawn, centrifuged at 1,000 x g for 20 min at 4 °C, and serum was collected. Serum insulin-like growth factor-1(IGF-1) trends were measured using an ELISA kit (R&D Systems, SMG100).

#### 2.2.3 Blood lipid profile measurement

On day 21, five male pups per group were fasted overnight, and blood was collected via cardiac puncture. After clotting at room temperature for 20 min, samples were centrifuged at 1,000 x g for 20 min at 4 °C to obtain serum. Serum triglycerides (TG), total cholesterol (TCHO), and high-density lipoprotein cholesterol (HDL-C) were measured using a FUJIFILM DRI-CHEM NX700i analyzer, with 10% samples randomly re-measured to confirm reliability.

### 2.3 Intraperitoneal injection of rhIGF-1 to intervene in EUGR

A recombinant human insulin-like growth factor-1 (rhIGF-1) intervention group and a PBS control group were established, with each EUGR dam nursing 10 pups. rhIGF-1 (Beyotime Biotechnology, Cat# P7489) was dissolved in PBS to a stock concentration of 100 μg/mL and stored at −80 °C. Immediately before use, the stock was diluted in PBS to a working concentration of 2.5*μ*g/mL. From postnatal day 8 to P21, pups received daily intraperitoneal injections of rhIGF-1 at 0.05μg/g body weight (0.02 mL/g) or an equal volume of PBS. Body weight, organ weights, and serum lipid profiles were assessed at the indicated time points (PND15 and PND21). Blood samples for serum IGF-1 measurement were collected at baseline (P8, prior to the first injection) and on P15 and P21.

### 2.4 Transcriptome analysis

Liver specimens from 3-week-old male pups in the Ctrl, EUGR, and EUGR (rhIGF1) groups were used for mRNA microarray analysis. For each group, liver samples from *n* = 2 individual pups (each pup from a distinct litter) were collected and processed independently, resulting in a total of 6 samples. Microarray experiments were performed on the Agilent platform by the Aksomics company. Briefly, total RNA was labeled using the Agilent Quick Amp Labeling Kit and hybridized to the microarrays using an Agilent SureHyb hybridization chamber. After washing, the arrays were scanned with an Agilent DNA Microarray Scanner, and raw signal intensities were extracted using Agilent Feature Extraction software (v11.0.1.1). Quantile normalization and subsequent data processing were performed using GeneSpring GX v12.1 software (Agilent Technologies). After normalization, raw data were filtered for high-quality probes for further analysis.

Differentially expressed genes (DEGs) between groups were identified based on the Benjamini-Hochberg corrected *p*-value (i.e., false discovery rate, FDR). The thresholds were set at Fold Change ≥ 1.5 (|log2FC| > 0.585) and FDR ≤ 0.05. To account for the inherent biological heterogeneity of *in vivo* tissue and the exploratory nature of the study with *n* = 2 per group, a moderate FC cutoff of 1.5 was adopted to capture moderate yet biologically relevant transcriptional changes. Statistically significant DEG groups were visualized using volcano plots. Hierarchical clustering was performed using an R script. Pathway analysis was conducted using standard enrichment calculation methods. KEGG pathways are displayed as Pathway Maps, illustrating the network of genes (proteins), small molecules, etc., involved in each pathway's function. The differentially expressed genes were used for pathway analysis to infer the pathways in which they are involved.

### 2.5 RT-PCR of differential pathway *PPARα*/*LPL*/*CD36*

Total RNA was isolated from male pup liver tissues (*n* = 5) using Trizol reagent. RNA was reverse transcribed into cDNA with SuperScript III Reverse Transcriptase. Quantitative PCR was performed on a QuantStudio5 system. Each 10 μL reaction contained cDNA, 200 μM primers, and Master Mix. The protocol was: 95 °C for 10 min; 40 cycles of 95 °C for 10 s, 60 °C for 60 s Gene expression was calculated via the standard curve method, with *β*-actin as the internal reference.

### 2.6 Cell experiments

#### 2.6.1 Cell culture and transfection

The human hepatocellular carcinoma cell line HepG2 used in this experiment was generously provided by the Institute of Cardiovascular Sciences, Peking University. The recombinant pcDNA3.1(+)-human *LPL*-3Xflag plasmid and empty vector were transfected into HepG2 cells as the experimental and Ctrl groups, respectively. After 48 h, RNA and protein were extracted. qPCR and Western blotting were performed to detect *LPL* mRNA and protein expression levels.

#### 2.6.2 Cell proliferation assay

Cell proliferation in the experimental and Ctrl groups was detected using the EdU incorporation assay.

#### 2.6.3 Transcriptome analysis and validation

Transcriptome analysis was performed on experimental and control cells (*n* = 3) to identify differentially expressed genes and infer *LPL*-related lipid metabolism pathways. After a 48-hour culture, RNA was extracted, reverse transcribed, and qPCR was performed to detect the relative mRNA levels of differential genes.

### 2.7 Statistical analysis

For each litter, the mean values of all parameters from three pups were calculated, and the litter was used as the independent unit (Ctrl group, *n* = 3 litters; EUGR group, *n* = 3 litters). Data are presented as mean ± standard deviation (SD). Differences between groups were evaluated with independent samples t-tests; Welch's t-test was applied when Levene's test indicated unequal variances (*p* < 0.05). On account of body size differences, organ/body weight ratios (organ weight ÷ 3-week body weight   ×   100%) were computed and compared using independent t-tests. Given the number of comparisons, Bonferroni correction was employed: *α* = 0.005 for 10 original variables and *α* = 0.0083 for six ratio variables. Effect sizes are reported as Cohen's d (thresholds: 0.2, small; 0.5, medium; 0.8, large).

For comparisons among the four groups [Ctrl, EUGR, EUGR(PBS), EUGR (rhIGF1)] in body weight, length, organ weights, and organ/body ratios, one-way ANOVA followed by Tukey's HSD test was used, with litter as the unit. Serum IGF-1 levels, measured from different pups at each time point, were analyzed with repeated-measures ANOVA: time (10 postnatal days) as the within-subject factor, group (Ctrl vs. EUGR) as the between-subject factor, and the time   ×   group interaction to assess dynamic differences. Bonferroni correction was applied across time points, and the effect size was expressed as partial *η*^2^.

Body weight and length data were fitted with linear mixed models (LMMs) to account for repeated measures within pups and clustering within litters. Fixed effects included group, time, and their interaction; litter was a random intercept, and a first-order autoregressive covariance structure modeled repeated measurements. Denominator degrees of freedom were estimated via Satterthwaite approximation, with *post hoc* comparisons adjusted by Bonferroni correction. Serum lipid profiles (TG, TCHO, HDL-C) were compared using one-way ANOVA and Tukey's HSD (litter as unit; *n* = 3 litters/group). A two-tailed *p* < 0.05 was considered significant. All analyses were performed in SPSS 26.0 (IBM Corp., Armonk, NY, USA).

## 3 Results

### 3.1 Characteristics of the EUGR model

#### 3.1.1 Growth characteristics of EUGR

During the experimental period, daily weighing of the maternal feed revealed that dietary intake varied considerably among dams of different body weights on the same postpartum day. Furthermore, the dietary intake of dams gradually increased over time postpartum, with significant variations (Appendix Table 1). Therefore, the feeding amount for dams in the EUGR group was matched based on maternal body weight and postpartum day.

**Figure 1:**
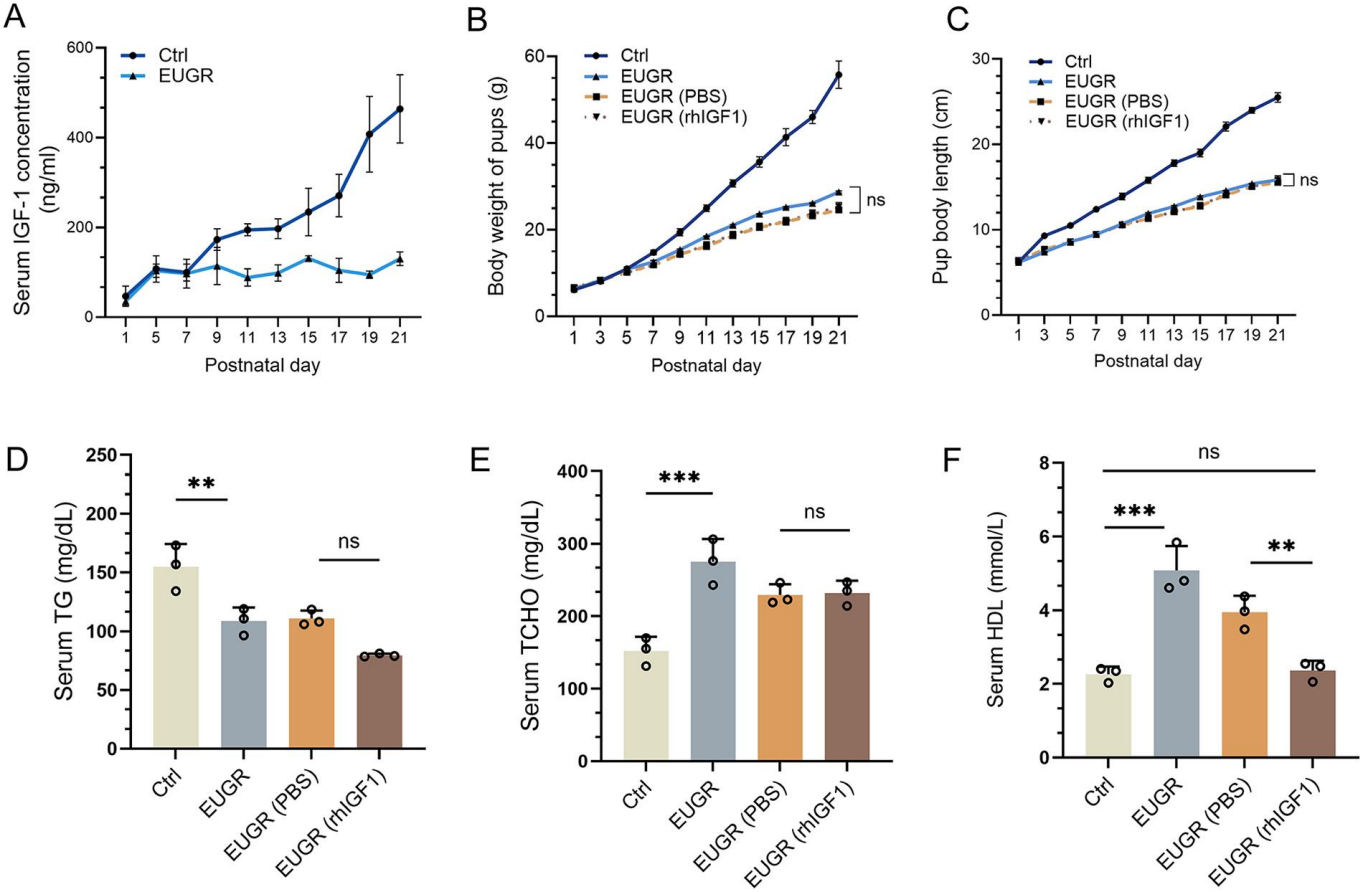
**(A)** trend of serum IGF-1 content in pups of the ctrl group (*n* = 3 litters) and EUGR group (*n* = 3 litters); the trends of IGF-1 content in the two groups were basically consistent in the first 7 days, while the IGF-1 content in the ctrl group continued to increase from 7 to 21 days compared with the EUGR group. **(B)** Average body weight of pups. No significant difference in weight gain was observed among the three groups, except the Ctrl group. **(C)** Average length of pups. The trend of change among groups is the same as that of average body weight. **(D–F)** Blood lipid indices of pups. Serum triglyceride (TG) levels (mg/dL) were significantly decreased in the EUGR group compared with the Ctrl group (*p* < 0.01). Total cholesterol (TCHO) levels (mg/dL) were significantly increased in all EUGR-related groups (*p* < 0.01). High-density lipoprotein cholesterol (HDL-C) levels (mmol/L) were elevated in the EUGR group (*p* < 0.001) but restored to near-control levels in the EUGR-IGF1 group (*p* = 0.992). Data are presented as mea*n* ± SD (*n* = 3 litters). One-way ANOVA followed by Tukey's HSD test was performed. *p < 0.05, **p < 0.01, ***p < 0.001 compared with the Ctrl group; ns indicates no significant difference.

As shown in Table 1, although the birth weight and length of male pups were similar between the two groups (*p* = 0.284 and *p* = 0.818, respectively), However, at 3 weeks of age, the EUGR group showed significantly lower body weight (28.72 ± 0.23 g vs. 53.66 ± 3.44 g, corrected *p* < 0.001), representing approximately 53.5% of the Ctrl group. Concurrently, the body length of the EUGR group (15.84 ± 0.18 cm) was also significantly shorter (corrected *p* < 0.001), about 62.1% of the Ctrl group. Weight differences emerged after day 7, while length growth rates diverged significantly from postnatal day 3 (Figures 1B,C). Additionally, the weights of all major organs in the EUGR group were lower (corrected *p* ≤ 0.002), with large effect sizes (Cohen's d > 5.6). Muscle showed the greatest reduction, dropping by 55.9% (44.1% of Ctrl; 0.15 g vs. 0.34 g), whereas the brain showed the smallest reduction, decreasing by only 8.7% (91.3% of Ctrl; 1.35 g vs. 1.48 g). To determine whether organ changes reflected active atrophy or passive scaling with body weight, organ/body weight ratios were analyzed (Table 1). The results demonstrated that the brain/body weight ratio was significantly higher in the experimental group compared to the Ctrl group (*p* < 0.001). Similarly, the tibia/body weight ratio showed a marked increase (*p* < 0.001), indicating a pronounced sparing effect on both brain and bone tissues under systemic growth-restricted conditions. In contrast, the muscle/body weight ratio was significantly reduced (*p* = 0.005), suggesting selective atrophy of muscular tissue. No statistically significant differences were observed in the weight ratios of the liver, heart, or kidneys between the two groups (all adjusted *p*-values > 0.0083), implying that changes in these organs primarily followed the overall reduction in body weight. These results indicate that maternal dietary restriction during lactation induces EUGR, characterized by reduced body weight, stunted growth, and decreased organ weights, though the degree of reduction varied among organs.

**Table 1:** The growth characteristics of neonate rats in group of ctrl and EUGR.

Indicators	Ctrl (*n* = 3)	EUGR (*n* = 3)	Test statistic	*P* value^a^	Cohen's *d* *d* *d*
Birthweight(g)	6.10 ± 0.12	6.32 ± 0.29	−1.24	0.284	−1.009
weight at 3 weeks(g)^‡^	53.66 ± 3.44	28.72 ± 0.23	12.52	**<0**.**001**	10.224
birth length(cm)	6.21 ± 0.04	6.18 ± 0.19	0.245	0.818	0.200
length at 3 weeks(cm)	25.50 ± 0.55	15.84 ± 0.18	28.90	**<0**.**001**	23.599
liver weight(g) ^b^	2.20 ± 0.20	1.06 ± 0.02	9.71	**<0**.**001**	7.925
liver body ratio (%)	4.09 ± 0.16	3.71 ± 0.04	3.96	0.017	3.24
heart weight(g)	0.38 ± 0.02	0.19 ± 0.01	17.05	**<0**.**001**	13.924
heart body ratio (%)	0.71 ± 0.02	0.67 ± 0.05	1.37	0.242	1.12
brain weight(g)	1.48 ± 0.03	1.35 ± 0.02	7.09	**0**.**002**	5.789
brain body ratio (%)	2.76 ± 0.15	4.70 ± 0.06	−21.43	**<0**.**001**	−17.49
kidney weight(g)	0.35 ± 0.04	0.19 ± 0.01	6.86	**0**.**002**	5.603
kidney body ratio (%)	0.66 ± 0.03	0.67 ± 0.04	−0.04	0.974	−0.03
muscle weight(g)	0.34 ± 0.01	0.15 ± 0.01	22.47	**<0**.**001**	18.346
muscle body ratio (%)	0.63 ± 0.02	0.52 ± 0.03	5.49	**0**.**005**	4.48
tibia weight(g)	0.27 ± 0.02	0.19 ± 0.01	7.14	**0**.**002**	5.83
tibia body ratio (%)	0.50 ± 0.01	0.67 ± 0.02	−15.56	**<0**.**001**	−12.7

Data are presented as mean ± SD of litter-averaged values (*n* = 3 litters per group).

a Bonferroni correction for ten comparisons: significance threshold *α*=0.005(0.05/10). **Bold** indicates statistical significance after correction.

b Unequal variances (Levene's test, p<0.05); Welch's *t* -test was used, and corrected degrees of freedom are reported.

Serum IGF-1 levels reflect growth status. Compared to the Ctrl group, IGF-1 concentrations in the EUGR group showed little difference within the first 7 postnatal days, being essentially the same on day 7 (97.93 ± 27.03 ng/mL vs. 99.52 ± 34.04 ng/mL, *p* = 0.94). However, after day 7, IGF-1 levels in the Ctrl group continued to rise, reaching about 463.94 ± 12.10 ng/mL by day 21 (Figure 1A). In contrast, EUGR group levels fluctuated around 100 ng/mL with minimal change. Repeated-measures ANOVA revealed a significant time   ×   group interaction for serum IGF-1 levels [F (9, 36) = 21.36, *p* < 0.001, *η*^2^ = 0.842], indicating that the dynamic changes in IGF-1 differed significantly between the Ctrl and EUGR groups. Bonferroni-corrected post-hoc comparisons (Table 2) showed that IGF-1 levels in the EUGR group were significantly lower than those in the Ctrl group from postnatal day 11 through day 21 (*p* < 0.05), whereas no significant differences were observed at days 0, 5, 7, and 9 (*p* > 0.05). These results demonstrate that pups under maternal dietary restriction have significantly lower serum IGF-1. Meanwhile, the temporal trend aligns with growth indicators, with differences emerging from day 7, suggesting an association between IGF-1 and growth and confirming growth restriction.

**Table 2:** Pairwise comparisons of serum IGF-1 levels between ctrl and EUGR groups (postnatal days11–21).

Postnatal day	Mean difference (Ctrl – EUGR)	SE	95% CI	*p* value^a^
11	106.24	13.54	[68.74, 143.74]	0.001
13	98.99	16.49	[53.20, 144.78]	0.004
15	103.29	30.68	[18.12, 188.46]	0.028
17	166.40	31.27	[79.59, 253.20]	0.006
19	313.10	48.87	[177.40, 448.79]	0.003
21	333.58	44.74	[209.37, 457.78]	0.002

a Bonferroni correction was applied for multiple comparisons across 10 time points. Adjusted *p*-values are shown; a two-tailed *p* < 0.05 was considered statistically significant. Non-significant differences were observed at days 0, 5, 7, and 9 (*p* > 0.05; data not shown).

#### 3.1.2 Lipid metabolism characteristics of EUGR rats

As shown in Table 3, for the comparison between Ctrl and EUGR groups, serum TG levels were significantly lower in the EUGR group (108.75 ± 11.40 mg/dL) than in the Ctrl group (154.75 ± 19.47 mg/dL, *p* < 0.01). Serum TCHO levels were significantly higher in the EUGR group (275.00 ± 31.56 mg/dL vs. 152.00 ± 19.50 mg/dL, *p* < 0.01). Similarly, HDL-C levels were significantly elevated in the EUGR group (5.08 ± 0.66 mmol/L vs. 2.26 ± 0.20 mmol/L, *p* < 0.01). These findings indicate that EUGR pups exhibit a lipid profile characterized by significantly elevated TCHO and HDL-C, as well as significantly reduced TG, suggesting the presence of underlying lipid metabolism alterations in EUGR.

**Table 3:** The lipid characteristics of neonate rats are born after 3 weeks in four groups.

Group	TG (mg/dL)	TCHO (mg/dL)	HDL-C (mmol/l)
EUGR(rhIGF-1) (*n* = 3)	79.50 ± 9.60*	232.00 ± 10.00*	2.36 ± 0.20
EUGR(PBS) (*n* = 3)	110.83 ± 9.34*	229.50 ± 14.40*	3.95 ± 0.56*
EUGR (*n* = 3)	108.75 ± 11.40*	275.00 ± 31.56*	5.08 ± 0.66*
Ctrl (*n* = 3)	154.75 ± 19.47	152.00 ± 19.50	2.26 ± 0.20
F (3,8)	20.81	16.92	29.18
*p*-value	<0.001	<0.001	<0.001

Data are presented as mean ± SD of litter-averaged values (*n* = 3 litters per group). One-way ANOVA followed by Tukey's HSD test was performed for each parameter.

TG, triglyceride; TCHO, total cholesterol; HDL-C, high-density lipoprotein.

* *p* < 0.05 compared with the Ctrl group.

### 3.2 rhIGF-1 Has No effects on growth of EUGR pups

Based on the IGF-1 results, since serum IGF-1 levels in the two groups of pups were similar during the first 7 days, daily intraperitoneal injections of rhIGF-1 or PBS began on postnatal day 8. Serum IGF-1 levels are shown in Table 4. On days 15 and 21, the EUGR(rhIGF1) group had significantly higher levels than the EUGR(PBS) group (*p* < 0.05). On day 15, the EUGR(rhIGF1) group's level (227.79 ± 23.27 ng/mL) was comparable to the Ctrl group's (234.42 ± 52.93 ng/mL), but on day 21, it was significantly lower (*p* < 0.001). This suggests that exogenous rhIGF-1 can be absorbed and increase systemic levels, but losses may occur, leading to a mismatch between the administered dose and the final serum increase.

**Table 4:** Serum IGF-1 concentrations at postnatal days 7, 15, and 21 (ng/mL).

Group	Day7	Day15	Day21
EUGR(rhIGF-1) (*n* = 3)	97.07 ± 9.26	227.79 ± 23.27	247.80 ± 14.56
EUGR(PBS) (*n* = 3)	97.54 ± 11.83	129.44 ± 20.58**	134.96 ± 11.98***
EUGR (*n* = 3)	97.93 ± 27.03	131.13 ± 4.82**	130.13 ± 11.08***
Ctrl (*n* = 3)	99.52 ± 34.04	234.42 ± 52.93	463.94 ± 12.10***
F (3,8)	0.02	15.85	52.92
*p*-value	0.99	<0.01	<0.001

Data are presented as mean ± SD of litter-averaged values (*n* = 3 litters per group).

IGF-1, insulin-like growth factor 1;

** *p* < 0.01

*** *p* < 0.001 compared with the EUGR(rhIGF-1) group.

As shown in Figures 1B,C, the growth trends in body weight and length between the rhIGF-1-treated group and the EUGR (PBS) group were largely consistent (*p* > 0.05) throughout the observation period. Linear mixed models were used to analyze body weight and body length, with group, time, and their interaction as fixed effects and litter as a random intercept (Table 5). A significant group   ×   time interaction was found for both body weight [F (30, 278.3) = 86.73, *p* < 0.001] and body length [F (30, 278.1) = 146.44, *p* < 0.001], confirming that the growth trajectories of the four groups differ significantly. However, post-hoc comparisons with Bonferroni correction revealed no significant differences between the EUGR(PBS) and EUGR(rhIGF1) groups at any time point for either body weight or body length, indicating that rhIGF-1 treatment did not significantly rescue the growth restriction phenotype under the current experimental conditions. The therapeutic effects comparison indicates that exogenous rhIGF-1 administration did not promote growth beyond the effect observed in the vehicle (PBS)-injected group.

**Table 5:** Fixed effects from linear mixed models for body weight and body length.

Dependent variable	Effect	Numerator df	Denominator df	F value	*p* value
Body weight	group	3	8.32	114.6	< 0.001
	time	10	278.32	1,120.93	< 0.001
	group × time	30	278.32	86.73	< 0.001
Body length	group	3	8.12	503.96	< 0.001
	time	10	278.12	3,080.65	< 0.001
	group × time	30	278.12	146.44	< 0.001

Linear mixed models were fitted with group, time, and their interaction as fixed effects, and litter as a random intercept. The denominator degrees of freedom were estimated using the Satterthwaite approximation. *p*-values indicate statistical significance at *α* < 0.05.

To assess the potential impact of the injection procedure itself, we compared both injected groups (rhIGF1 and PBS) to the uninjected EUGR group. After day 7, the growth curves of the EUGR(rhIGF1) and EUGR(PBS) groups were slightly lower than that of the EUGR group, suggesting that the injection procedure may have introduced stress that modestly impaired growth. For instance, on day 15, the body weight in the EUGR(rhIGF1) group (20.7 ± 2.49 g) was not significantly different from the EUGR group (23.7 ± 0.69 g) (*p* = 0.05), while length remained shorter (12.86 ± 0.66 cm vs. 13.9 ± 0.25 cm, *p* < 0.05). Therefore, the absence of a growth-promoting effect of rhIGF-1 is evident from its lack of difference from the EUGR(PBS) group, which was subjected to the same handling stress. Although exogenous rhIGF-1 elevated serum IGF-1 concentrations, it did not improve body weight or length compared to the Ctrl. We conclude that under the conditions of this study, including the potential stress of the administration method, rhIGF-1 did not ameliorate the established growth restriction in these pups.

### 3.3 rhIGF-1 improves the blood lipids of EUGR pups

One-way ANOVA demonstrated significant differences among the four groups in serum levels of TG [F (3,8) = 20.81, *p* < 0.001], TCHO [F (3,8) = 16.92, *p* < 0.001], and HDL [F (3,8) = 29.18, *p* < 0.001] levels. Tukey's post-hoc test showed that the EUGR and EUGR(PBS) groups had significantly lower TG levels than the Ctrl group (*p* < 0.01), while the EUGR(rhIGF1) group exhibited the lowest TG levels compared to the Ctrl group (*p* < 0.001). For TCHO, all three EUGR-related groups showed significantly higher levels than the Ctrl group (*p* < 0.01), with no significant difference between the EUGR(PBS) and EUGR(rhIGF1) group (*p* > 0.05). Notably, HDL levels in the EUGR(rhIGF1) group were significantly lower than those in the EUGR and EUGR(PBS) group (*p* < 0.01) and were comparable to those in the Ctrl group (*p* > 0.05). Collectively, these results indicate that rhIGF-1 can lower the elevated HDL-C induced by dietary restriction, suggesting a corrective effect.

### 3.4 rhIGF-1 effects on lipid metabolism related genes and pathways

**Figure 2:**
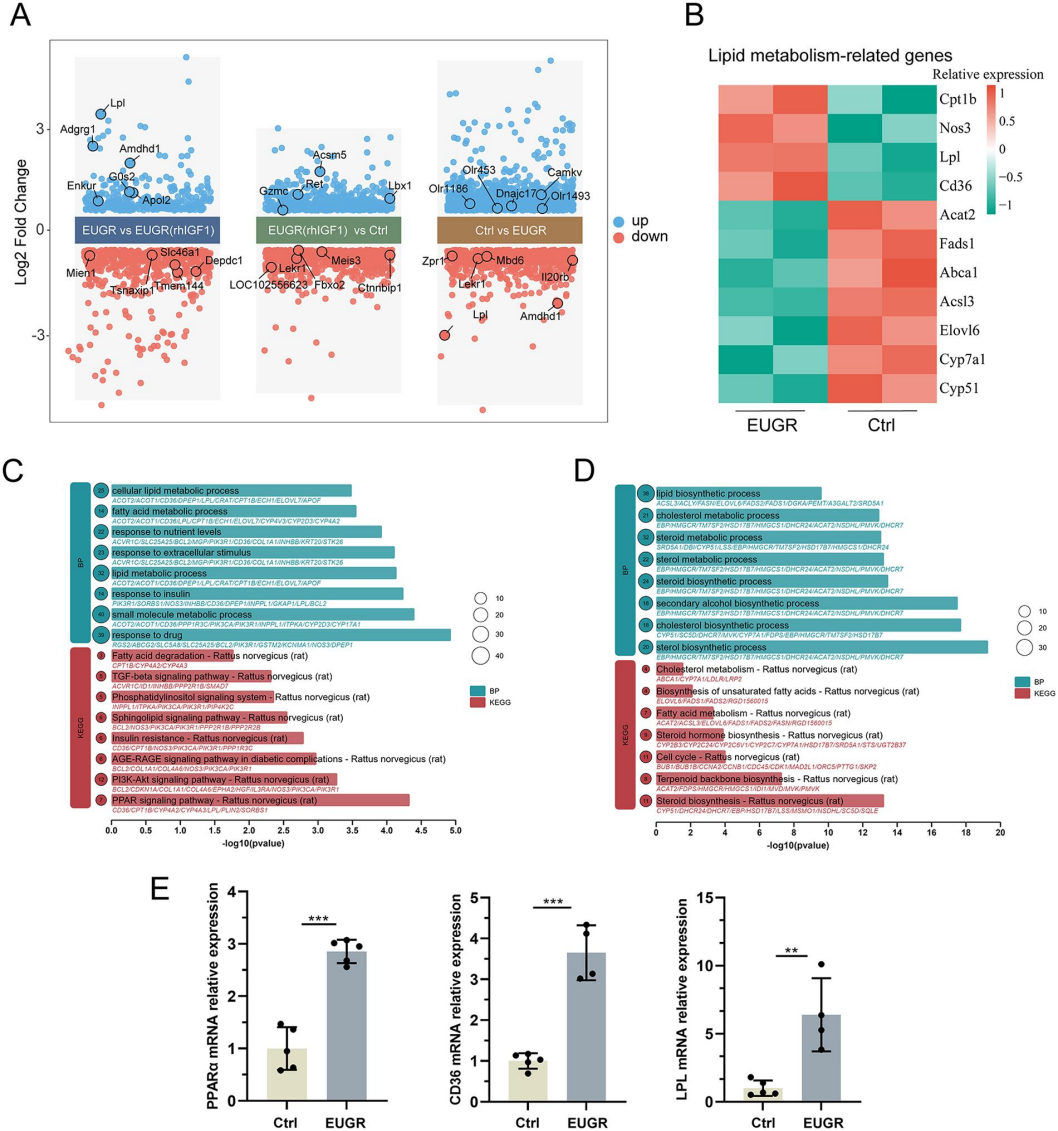
**(A)** volcano plot showing the distribution of differentially expressed genes between the ctrl group (*n* = 2) and EUGR group (*n* = 2). **(B)** Cluster heatmap; red indicates upregulation, blue indicates downregulation. **(C)** Pathways upregulated in the EUGR group (*n* = 2) compared to the Ctrl group(*n* = 2). **(D)** Pathways downregulated in the EUGR group (*n* = 2) compared to the Ctrl group(*n* = 2). **(E)** Gene expression levels of the *LPL*/*CD36*/*PPARα* pathway (**p < 0.01, ***p < 0.001).

To elucidate the regulatory mechanism of rhIGF-1 on lipid metabolism in rats with food restriction, we performed microarray-based transcriptomic profiling on liver tissues from Ctrl, EUGR, and EUGR (rhIGF1) groups. The volcano plot (Figure 2A) revealed 531 up- and 684 down-regulated mRNAs in EUGR vs. EUGR (rhIGF1) (FC ≥ 1.5; FDR ≤ 0.05), while EUGR (rhIGF1) vs. Ctrl showed markedly fewer DEGs (318 up, 572 down), indicating that rhIGF1 promoted transcriptomic restoration. Ctrl vs. EUGR displayed 1,016 up-regulated and 657 down-regulated mRNAs. *LPL* was identified among the down-regulated genes in this comparison, indicating that its expression was higher in the EUGR group relative to Ctrl. Consistently, *LPL* was up-regulated in EUGR and restored by rhIGF-1, suggesting *LPL* as a key regulator. Meanwhile, in Heatmap analysis (Figure 2B), reduced expression of lipid metabolism-related genes (e.g., *LPL*: log₂FC = –3.24, FDR = 0.01; *CD36*: log₂FC = –0.85, FDR = 0.02) in Ctrl, with higher levels in EUGR.

GO/KEGG enrichment showed up-regulated “lipid catabolic process” and “fatty acid metabolic process” and down-regulated “steroid biosynthesis,” “cholesterol metabolic process,” and “fatty acid degradation” in EUGR (Figures 2C,D). KEGG revealed activation of “*PPAR* signaling” and “*PI3K*-*Akt* signaling,” while “steroid biosynthesis” and “fatty acid metabolism” were suppressed. These results suggest rhIGF-1 ameliorates hepatic lipid disorder by modulating lipid metabolism, potentially via *LPL*.

### 3.5 Upregulation of *PPARα/LPL/CD36* expression affects Serum TG

Combined with the lipid metabolism characteristics in the Ctrl and EUGR group, the *PPAR* pathway was selected for RT-qPCR validation of the microarray results of *LPL*, *CD36*, and *PPARα* genes. RT-qPCR results were consistent with transcriptomic data (Figure 2E). Compared to Ctrl groups, the relative mRNA levels of *LPL*, *CD36*, and *PPARα* in the EUGR group were significantly increased (*p* < 0.05), indicating these differential genes may underlie the lipid metabolism abnormalities. Specifically, the relative expression of *LPL* in the EUGR group was 6.39 ± 2.68, significantly higher than in the Ctrl groups (*p* < 0.05), which, combined with serological data, suggests it may be a cause for the decreased serum TG.

### 3.6 Overexpression of *LPL* inhibits HepG2 cell proliferation

**Figure 3:**
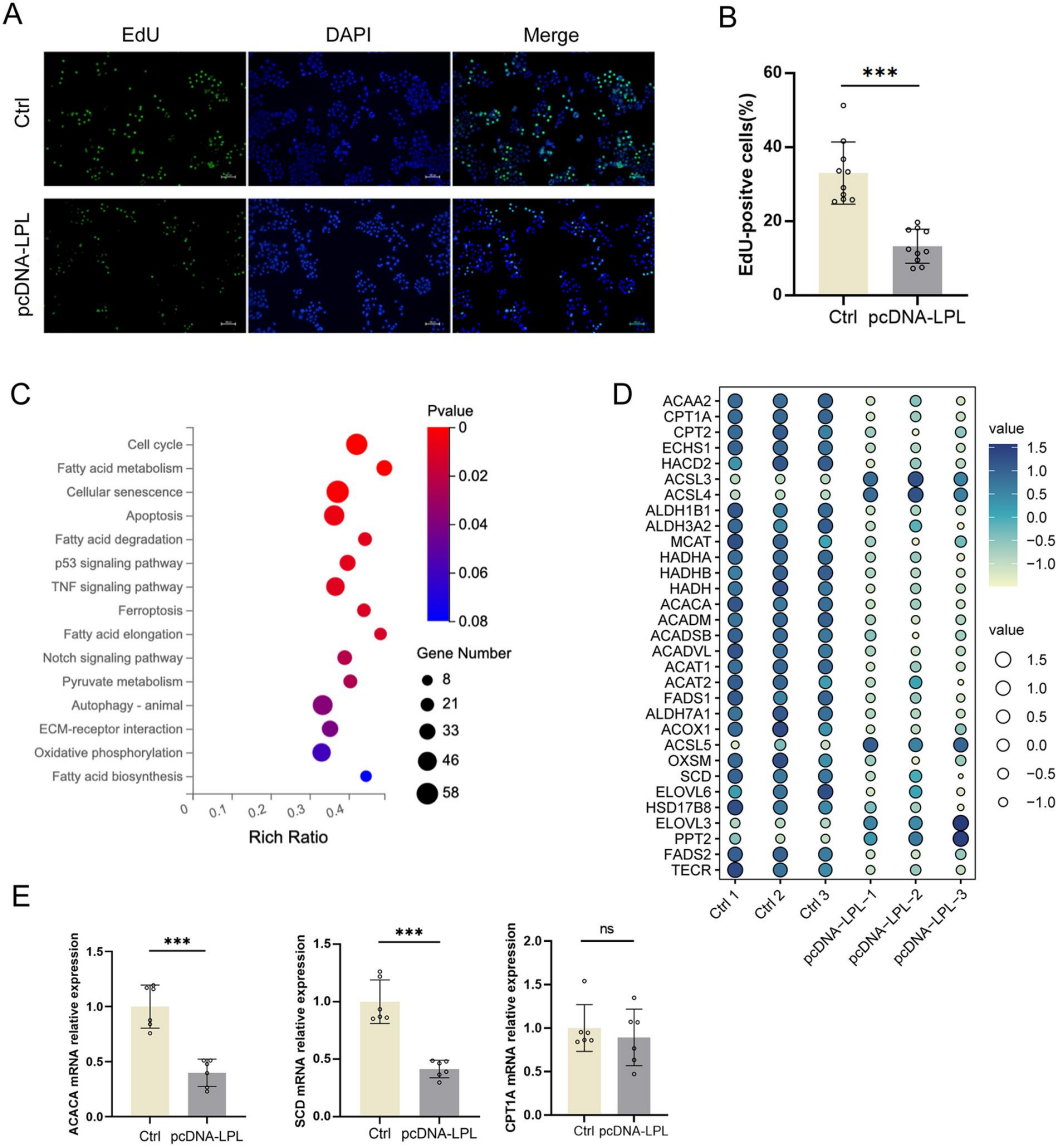
**(A)** cell proliferation ability in the experimental and ctrl group was assessed by EdU assay. **(B)** Quantitative results of the EdU assay for cells in the experimental and Ctrl group. **(C)** The results of KEGG pathway enrichment analysis. **(D)** Heatmap showing the expression levels of key genes related to lipid metabolism in the two groups. The color gradient from blue to white represents gene expression levels from high to low (blue indicates high expression; white indicates low expression). **(E)** Gene expression levels of the *ACACA*/*SCD*/*CPT1A* pathway (***p < 0.001).

To investigate the effect of *LPL*, an *LPL*-overexpressing cell line was generated in HepG2 cells. Forty-eight hours after transfection with pcDNA3.1(+)-human *LPL*-3Xflag, compared to the Ctrl group, the *LPL* mRNA expression in the experimental group was significantly increased (*p* < 0.01), by 3.0 × 10^4^-fold. EdU assay results showed that at 48 h post-transfection, compared to the Ctrl group, the number of EdU-positive cells in the experimental group decreased, indicating inhibited cell proliferation capacity (*p* < 0.01, Figures 3A,B).

### 3.7 The lipid metabolism genes *ACACA* and *SCD* are affected by *LPL* overexpression

Transcriptome analysis of experimental and control cells revealed 538 mRNAs with altered expression in the experimental group compared to controls, comprising 421 upregulated and 117 downregulated (FC ≥ 2.0; FDR ≤ 0.05). A more stringent FC cutoff was used for this homogeneous cell line to focus on pronounced transcriptional changes. KEGG enrichment analysis (Figure 3C) identified lipid metabolism-related pathways, including Fatty acid metabolism, degradation, and elongation, involving 32 lipid metabolism genes. By comparing the differential gene heatmap (Figure 3D) and considering the effect of *LPL* on TG, the genes *CPT1A*, *ACACA*, and *SCD* were selected for validation. RT-qPCR results (Figure 3E) showed that relative mRNA levels of *ACACA* and *SCD* were significantly reduced in the experimental group (*p* < 0.01), with no difference for *CPT1A*. These results indicate that LPL overexpression alters *ACACA* and *SCD* expression, which may underlie the *LPL*-induced TG reduction.

## 4 Discussion

In this study, we simulated EUGR by limiting maternal dietary intake during lactation, leading to offspring with reduced body weight, shortened body length, and decreased organ-to-body weight ratios. Fasting and delayed full enteral nutrition are independent risk factors for EUGR (35, 36). Studies employing a 50% dietary restriction in lactating dams support our model (20, 21). EUGR group male pups showed significantly lower body weight (53.7% of Ctrl groups, *p* < 0.01) and reduced serum IGF-1 levels (*p* < 0.01), confirming growth restriction. Maternal dietary protein restriction reduces breast milk protein, fat, and free amino acids (22), which are positively correlated with weight gain (37). This alteration in breast milk composition may contribute to impaired offspring growth and metabolic changes.

Based on the results showing no difference in body weight and body length growth between the rhIGF-1-treated group and the PBS control group, exogenous rhIGF-1 may not ameliorate EUGR. Although IGF-1 is a major growth hormone promoting muscle and bone growth (38, 39) and has shown efficacy in specific contexts (40–44), a phase II RCT found it did not improve retinopathy progression (45), and a study in preterm piglets showed no significant weight gain, consistent with our findings (46). Given the limited number of studies, we hypothesize that the lack of effect may be due to insufficient serum IGF-1 levels, its short half-life, or discontinuous administration (47). Additionally, the use of intraperitoneal injection as the route of administration for rhIGF-1 might be one of the limitations, while necessary for controlled delivery, it introduced a handling stress that likely confounded the assessment of its pure effect on growth. Future studies employing less invasive or stress-minimizing delivery methods (e.g., subcutaneous osmotic pumps, oral supplementation via milk) would help clarify the true therapeutic potential of rhIGF-1 in this model.

In addition to growth restriction, the EUGR group exhibited significant lipid metabolism abnormalities, characterized by low triglycerides, high total cholesterol, and high HDL-C, consistent with previous findings (21). Transcriptomic analysis revealed alterations in lipid metabolism-related pathways, such as *PPARα* and Fatty acid metabolism. The high TCHO and HDL-C in the EUGR group warrant consideration. While sustained energy restriction is often linked to lower TCHO and LDL-C (48, 49), studies report elevated HDL-C (50) and TCHO (51) under calorie restriction. We speculate the elevated TCHO may be due to impaired liver function or a compensatory stress response to growth restriction. Triglycerides (TG) are critical for fetal and neonatal development, with low levels adversely affecting growth. Maternal dietary restriction during lactation is a potential cause of low pup TG. Transcriptomic analysis suggests TG levels are associated with *PPARα*, *CD36*, and *LPL* alterations. *PPARα* activation stimulates *LPL* expression, reducing TG (52). Cell experiments show *PPARα* enhances *LPL* mRNA stability and expression (53), and rat studies confirm that increased hepatic *PPARα* elevates perinatal *LPL* activity (54). *CD36* facilitates free fatty acid uptake; its deficiency elevates plasma TG, partly due to reduced *LPL* activity (55–57). *LPL* is a key regulator of blood and hepatic TG (58, 59). The increased *LPL* expression in the EUGR liver is likely to contribute to reduced TG levels.

IGF-1 is linked to lipid metabolism (60). Using the Ctrl group as a baseline to analyze changes in lipid metabolic indices, our results showed that rhIGF-1 restored HDL-C levels in the EUGR group to those of the Ctrl group, suggesting a positive ameliorative effect on EUGR. However, it had no significant impact on TCHO. The further reduction in TG observed aligns with findings from studies showing that high-dose rhIGF-1 can lower fasting TG levels in healthy adults (61).

This study further investigated the mechanism of TG reduction via *LPL* overexpression in HepG2 cells. *LPL* overexpression reduced cell proliferation. *LPL* hydrolyzes TG in lipoproteins, facilitating lipid uptake into tissues. In transgenic mice with muscle-specific *LPL* expression, decreased plasma TG, increased muscle free fatty acid uptake, muscle atrophy, weight loss, and premature death occurred due to excessive lipid accumulation in muscle cells. Ectopic lipid accumulation can induce apoptosis and muscle damage (62). We hypothesize that *LPL* overexpression similarly causes excessive TG accumulation in HepG2 cells, triggering apoptosis and reduced proliferation.

The HepG2 cell line is widely used for studying lipid metabolism. This research explored the lipid metabolism-related genes altered by *LPL* overexpression in HepG2 cells. Transcriptomic analysis and RT-qPCR validation revealed a significant decrease in *ACACA* and *SCD* mRNA in *LPL*-overexpressing cells. While no prior direct analysis of *ACACA* and *SCD* expression following *LPL* manipulation in this cell line exists, both genes are linked to feeding patterns and lipid metabolism. Dietary regimens influence *ACACA* levels; for instance, high-concentrate diets in goats reduce milk fat, mammary TG, and *ACACA/FASN* mRNA (63, 64). *ACACA* expression correlates with lipid deposition in muscle (65). *SCD*, the rate-limiting enzyme in monounsaturated fatty acid synthesis, is crucial in hepatic *de novo* lipogenesis. Enhanced *SCD* activity promotes hepatic TG accumulation, driving *NAFLD* progression. *PPARγ* activation upregulates lipogenic genes like *SCD* to promote TG synthesis in muscle (66). Dietary composition affects *SCD*; higher protein diets in weaned piglets reduced serum TG/TCHO, increased HDL, and decreased hepatic *SCD* mRNA (67). Our results indicate that *LPL* overexpression downregulates *ACACA* and *SCD*, suggesting *LPL* may influence lipogenesis. This provides a potential basis for the hypothesis that maternal dietary restriction during lactation could alter *ACACA* and *SCD* expression.

The implications of this study for humans are that it identifies a specific dyslipidemia profile and a key biological pathway (*LPL*/*CD36*/*PPARα*) linked to early undernutrition, providing clear targets for future research in preterm infants. It suggests that clinical care must prioritize optimal early nutrition to prevent long-term metabolic issues and that effective therapies, potentially including agents like rhIGF-1, may need to focus on correcting this underlying metabolic dysfunction rather than solely promoting weight gain.

## 5 Conclusion

This study successfully established an EUGR rat model and conducted transcriptomic analysis on the livers of EUGR rats. We further attempted to intervene in EUGR and its associated lipid metabolism disorder using rhIGF-1. The results demonstrate that EUGR in rats is primarily characterized by reduced body weight, shortened body length, and decreased weight of major organs. This condition was accompanied by a distinctive lipid metabolism disorder characterized by low triglycerides (TG), high total cholesterol (TCHO), and high high-density lipoprotein cholesterol (HDL-C). This metabolic abnormality may be associated with the *LPL/CD36/PPARα* pathway. Furthermore, while rhIGF-1 showed no clear improvement in growth restriction or the abnormalities in TG and TCHO levels, it effectively reduced the elevated HDL-C induced by EUGR.

## Data Availability

The data presented in the study are deposited in the NCBI Sequence Read Archive (SRA) repository, accession number PRJNA1470395.

## References

[1] Fenton TR Chan HT Madhu A Griffin IJ Hoyos A Ziegler EE. Preterm infant growth velocity calculations: a systematic review. Pediatrics. (2017) 139(3):e20162045. 10.1542/peds.2016-2045 28246339

[2] Fenton TR Cormack B Goldberg D Nasser R Alshaikh B Eliasziw M. Extrauterine growth restriction” and “postnatal growth failure” are misnomers for preterm infants. J Perinatol. (2020) 40(5):704–14. 10.1038/s41372-020-0658-5 32214217

[3] Clark RH Thomas P Peabody J. Extrauterine growth restriction remains a serious problem in prematurely born neonates. Pediatrics. (2003) 111(5 Pt 1):986–90. 10.1542/peds.111.5.986 12728076

[4] Fanaroff AA Stoll BJ Wright LL Carlo WA Ehrenkranz RA Stark AR. Trends in neonatal morbidity and mortality for very low birthweight infants. Am J Obstet Gynecol. (2007) 196(2):147.e1–8. 10.1016/j.ajog.2006.09.014 17306659

[5] Gidi NW Goldenberg RL Nigussie AK McClure E Mekasha A Worku B. Incidence and associated factors of extrauterine growth restriction (EUGR) in preterm infants, a cross-sectional study in selected NICUs in Ethiopia. BMJ Paediatr Open. (2020) 4(1):e000765. 10.1136/bmjpo-2020-000765 33094173 PMC7552851

[6] Ong KK Kennedy K Castañeda-Gutiérrez E Forsyth S Godfrey KM Koletzko B. Postnatal growth in preterm infants and later health outcomes: a systematic review. Acta Paediatr. (2015) 104(10):974–86. 10.1111/apa.13128 26179961 PMC5054880

[7] Ordóñez-Díaz MD Pérez-Navero JL Flores-Rojas K Olza-Meneses J Muñoz-Villanueva MC Aguilera-García CM. Prematurity with extrauterine growth restriction increases the risk of higher levels of glucose, low-grade of inflammation, and hypertension in prepubertal children. Front Pediatr. (2020) 8:180. 10.3389/fped.2020.00180 32373566 PMC7186313

[8] Ortiz Espejo M Gil Campos M Muñoz Villanueva MC Pérez Navero JL. [Metabolic changes in prepuberty children with extrauterine growth restriction]. An Pediatr (Barc). (2012) 77(4):247–53. 10.1016/j.anpedi.2012.02.011 22494944

[9] Desai M Jellyman JK Ross MG. Epigenomics, gestational programming and risk of metabolic syndrome. Int J Obes (Lond). (2015) 39(4):633–41. 10.1038/ijo.2015.13 25640766

[10] Lukaszewski MA Eberlé D Vieau D Breton C. Nutritional manipulations in the perinatal period program adipose tissue in offspring. Am J Physiol Endocrinol Metab. (2013) 305(10):E1195–207. 10.1152/ajpendo.00231.2013 24045869

[11] Drehmer M Duncan BB Kac G Schmidt MI. Association of second and third trimester weight gain in pregnancy with maternal and fetal outcomes. PLoS One. (2013) 8(1):e54704. 10.1371/journal.pone.0054704 23382944 PMC3559868

[12] Gillman MW. The first months of life: a critical period for development of obesity. Am J Clin Nutr. (2008) 87(6):1587–9. 10.1093/ajcn/87.6.1587 18541543 PMC4407661

[13] Meas T. Fetal origins of insulin resistance and the metabolic syndrome: a key role for adipose tissue? Diabetes Metab. (2010) 36(1):11–20. 10.1016/j.diabet.2009.09.001 19815442

[14] Singhal A Cole TJ Fewtrell M Deanfield J Lucas A. Is slower early growth beneficial for long-term cardiovascular health? Circulation. (2004) 109(9):1108–13. 10.1161/01.Cir.0000118500.23649.Df 14993136

[15] Pecks U Brieger M Schiessl B Bauerschlag DO Piroth D Bruno B. Maternal and fetal cord blood lipids in intrauterine growth restriction. J Perinat Med. (2012) 40(3):287–96. 10.1515/jpm.2011.135 22505508

[16] Roggero P Giannì ML Liotto N Taroni F Orsi A Amato O. Rapid recovery of fat mass in small for gestational age preterm infants after term. PLoS One. (2011) 6(1):e14489. 10.1371/journal.pone.0014489 21245927 PMC3016317

[17] Kim K Kim NJ Kim SY. Safety and efficacy of early high parenteral lipid supplementation in preterm infants: a systematic review and meta-analysis. Nutrients. (2021) 13(5):1535. 10.3390/nu13051535 34063216 PMC8147506

[18] Fischer CJ Maucort-Boulch D Essomo Megnier-Mbo CM Remontet L Claris O. Early parenteral lipids and growth velocity in extremely-low-birth-weight infants. Clin Nutr. (2014) 33(3):502–8. 10.1016/j.clnu.2013.07.007 23958274

[19] Alburaki W Yusuf K Dobry J Sheinfeld R Alshaikh B. High early parenteral lipid in very preterm infants: a randomized-controlled trial. J Pediatr. (2021) 228:16–23.e1. 10.1016/j.jpeds.2020.08.024 32798567

[20] Calkins KL Thamotharan S Dai Y Shin BC Kalhan SC Devaskar SU. Early dietary restriction in rats alters skeletal muscle tuberous sclerosis complex, ribosomal S6, and mitogen-activated protein kinase. Nutr Res. (2018) 54:93–104. 10.1016/j.nutres.2018.03.013 29685622 PMC6008232

[21] Lee S You YA Kwon EJ Jung SC Jo I Kim YJ. Maternal food restriction during pregnancy and lactation adversely affect hepatic growth and lipid metabolism in three-week-old rat offspring. Int J Mol Sci. (2016) 17(12):2115. 10.3390/ijms17122115 27983688 PMC5187915

[22] Eleftheriades M Pervanidou P Vafaei H Vaggos G Dontas I Skenderi K. Metabolic profiles of adult Wistar rats in relation to prenatal and postnatal nutritional manipulation: the role of birthweight. Hormones (Athens). (2014) 13(2):268–79. 10.1007/bf03401341 24776627

[23] Qasem RJ Li J Tang L Browne R Mynatt R McLeod C. Maternal protein restriction during pregnancy and lactation in rats imprints long-term reduction in hepatic lipid content selectively in the male offspring. Nutr Res. (2010) 30(6):410–7. 10.1016/j.nutres.2010.06.008 20650349

[24] Martin Agnoux A El Ghaziri A Moyon T Pagniez A David A Simard G. Maternal protein restriction during lactation induces early and lasting plasma metabolomic and hepatic lipidomic signatures of the offspring in a rodent programming model. J Nutr Biochem. (2018) 55:124–41. 10.1016/j.jnutbio.2017.11.009 29413487

[25] Czech MP. Obesity notches up fatty liver. Nat Med. (2013) 19(8):969–71. 10.1038/nm.3293 23921741

[26] Hellström A Ley D Hansen-Pupp I Hallberg B Löfqvist C Van Marter L. Insulin-Like growth factor 1 has multisystem effects on foetal and preterm infant development. Acta Paediatr. (2016) 105(6):576–86. 10.1111/apa.13350 26833743 PMC5069563

[27] Dominici FP Argentino DP Muñoz MC Miquet JG Sotelo AI Turyn D. Influence of the crosstalk between growth hormone and insulin signalling on the modulation of insulin sensitivity. Growth Horm IGF Res. (2005) 15(5):324–36. 10.1016/j.ghir.2005.07.001 16112592

[28] LeRoith D Yakar S. Mechanisms of disease: metabolic effects of growth hormone and insulin-like growth factor 1. Nat Clin Pract Endocrinol Metab. (2007) 3(3):302–10. 10.1038/ncpendmet0427 17315038

[29] Chiesa C Osborn JF Haass C Natale F Spinelli M Scapillati E. Ghrelin, leptin, IGF-1, IGFBP-3, and insulin concentrations at birth: is there a relationship with fetal growth and neonatal anthropometry? Clin Chem. (2008) 54(3):550–8. 10.1373/clinchem.2007.095299 18202160

[30] Randhawa R Cohen P. The role of the insulin-like growth factor system in prenatal growth. Mol Genet Metab. (2005) 86(1-2):84–90. 10.1016/j.ymgme.2005.07.028 16165387

[31] Kajantie E. Insulin-Like growth factor (IGF)-I, IGF binding protein (IGFBP)-3, phosphoisoforms of IGFBP-1 and postnatal growth in very-low-birth-weight infants. Horm Res. (2003) 60(Suppl 3:):124–30. 10.1159/000074513 14671409

[32] Orbak Z Darcan S Coker M Gökşen D. Maternal and fetal Serum insulin-like growth factor-I (IGF-I) IGF binding protein-3 (IGFBP-3), leptin levels and early postnatal growth in infants born asymmetrically small for gestational age. J Pediatr Endocrinol Metab. (2001) 14(8):1119–27. 10.1515/jpem-2001-0808 11592569

[33] Jaquiery AL Vickers MH. Editorial: understanding the impact on metabolic health of interactions between pre- and post-natal nutrition, sex, growth, and endocrine development. Front Endocrinol (Lausanne). (2024) 15:1503983. 10.3389/fendo.2024.1503983 39534258 PMC11554450

[34] Mao Y Zhao Y Luo S Chen H Liu X Wu T. Advanced paternal age increased metabolic risks in mice offspring. Biochim Biophys Acta Mol Basis Dis. (2022) 1868(5):166355. 10.1016/j.bbadis.2022.166355 35131436

[35] Lee SM Kim N Namgung R Park M Park K Jeon J. Prediction of postnatal growth failure among very low birth weight infants. Sci Rep. (2018) 8(1):3729. 10.1038/s41598-018-21647-9 29487306 PMC5829148

[36] Shen W Zheng Z Lin XZ Wu F Tian QX Cui QL. Incidence of extrauterine growth retardation and its risk factors in very preterm infants during hospitalization: a multicenter prospective study. Zhongguo Dang Dai Er Ke Za Zhi. (2022) 24(2):132–40. 10.7499/j.issn.1008-8830.2111143 35209977 PMC8884052

[37] Lin YH Hsu YC Lin MC Chen CH Wang TM. The association of macronutrients in human milk with the growth of preterm infants. PLoS One. (2020) 15(3):e0230800. 10.1371/journal.pone.0230800 32214387 PMC7098608

[38] Hawkins-Carranza FG Muñoz-Calvo MT Martos-Moreno GÁ Allo-Miguel G Del Río L Pozo J. rhIGF-1 treatment increases bone mineral density and trabecular bone structure in children with papp-A2 deficiency. Horm Res Paediatr. (2018) 89(3):200–4. 10.1159/000486336 29455208

[39] Chen J Smith LE. Retinopathy of prematurity. Angiogenesis. (2007) 10(2):133–40. 10.1007/s10456-007-9066-0 17332988

[40] Skarsgard ED Amii LA Dimmitt RA Sakamoto G Brindle ME Moss RL. Fetal therapy with rhigf-1 in a rabbit model of intrauterine growth retardation. J Surg Res. (2001) 99(1):142–6. 10.1006/jsre.2001.6120 11421616

[41] Backeljauw PF Miller BS Dutailly P Houchard A Lawson E Hale DE. Recombinant human growth hormone plus recombinant human insulin-like growth factor-1 coadministration therapy in short children with low insulin-like growth factor-1 and growth hormone sufficiency: results from a randomized, multicenter, open-label, parallel-group, active treatment-controlled trial. Horm Res Paediatr. (2015) 83(4):268–79. 10.1159/000371799 25765099

[42] Miller BS Duffy MM Addo OY Sarafoglou K. rhIGF-1 therapy for growth failure and IGF-1 deficiency in congenital disorder of glycosylation Ia (PMM2 deficiency). J Investig Med High Impact Case Rep. (2013) 1(3):2324709613503316. 10.1177/2324709613503316 26425584 PMC4586814

[43] Luque V Escribano J Grote V Ferre N Koletzko B Gruszfeld D. Does insulin-like growth factor-1 mediate protein-induced kidney growth in infants? A secondary analysis from a randomized controlled trial. Pediatr Res. (2013) 74(2):223–9. 10.1038/pr.2013.87 23708690

[44] Seedorf G Kim C Wallace B Mandell EW Nowlin T Shepherd D. rhIGF-1/Bp3 preserves lung growth and prevents pulmonary hypertension in experimental bronchopulmonary dysplasia. Am J Respir Crit Care Med. (2020) 201(9):1120–34. 10.1164/rccm.201910-1975OC 32101461 PMC7193843

[45] Ley D Hallberg B Hansen-Pupp I Dani C Ramenghi LA Marlow N. rhIGF-1/rhIGFBP-3 in preterm infants: a phase 2 randomized controlled trial. J Pediatr. (2019) 206:56–65.e8. 10.1016/j.jpeds.2018.10.033 30471715 PMC6389415

[46] Holgersen K Rasmussen MB Carey G Burrin DG Thymann T Sangild PT. Clinical outcome and gut development after insulin-like growth factor-1 supplementation to preterm pigs. Front Pediatr. (2022) 10:868911. 10.3389/fped.2022.868911 35989990 PMC9389362

[47] Hellström A Ley D Hallberg B Löfqvist C Hansen-Pupp I Ramenghi LA. Igf-1 as a drug for preterm infants: a step-wise clinical development. Curr Pharm Des. (2017) 23(38):5964–70. 10.2174/1381612823666171002114545 28969546 PMC5824464

[48] Meng H Zhu L Kord-Varkaneh H O Santos H Tinsley GM Fu P. Effects of intermittent fasting and energy-restricted diets on lipid profile: a systematic review and meta-analysis. Nutrition. (2020) 77:110801. 10.1016/j.nut.2020.110801 32428841

[49] Jayedi A Zeraattalab-Motlagh S Jabbarzadeh B Hosseini Y Jibril AT Shahinfar H. Dose-dependent effect of carbohydrate restriction for type 2 diabetes management: a systematic review and dose-response meta-analysis of randomized controlled trials. Am J Clin Nutr. (2022) 116(1):40–56. 10.1093/ajcn/nqac066 35537861

[50] Ghanavati M Alipour Parsa S Nasrollahzadeh J. A calorie-restricted diet with nuts favourably raises plasma high-density lipoprotein-cholesterol in overweight and obese patients with stable coronary heart disease: a randomised controlled trial. Int J Clin Pract. (2021) 75(9):e14431. 10.1111/ijcp.14431 34080258

[51] Waldman HS Smith JEW Lamberth J Fountain BJ Bloomer RJ Butawan MB. A 28-day carbohydrate-restricted diet improves markers of cardiovascular disease in professional firefighters. J Strength Cond Res. (2020) 34(10):2785–92. 10.1519/jsc.0000000000003749 32740289

[52] Fruchart JC Staels B Duriez P. PPARs, metabolic disease and atherosclerosis. Pharmacol Res. (2001) 44(5):345–52. 10.1006/phrs.2001.0871 11712864

[53] Li L Beauchamp MC Renier G. Peroxisome proliferator-activated receptor alpha and gamma agonists upregulate human macrophage lipoprotein lipase expression. Atherosclerosis. (2002) 165(1):101–10. 10.1016/s0021-9150(02)00203-4 12208475

[54] Panadero M Bocos C Herrera E. Relationship between lipoprotein lipase and peroxisome proliferator-activated receptor-alpha expression in rat liver during development. J Physiol Biochem. (2006) 62(3):189–98. 10.1007/bf03168468 17451160

[55] Abumrad NA El-Maghrabi MR Amri EZ Lopez E Grimaldi PA. Cloning of a rat adipocyte membrane protein implicated in binding or transport of long-chain fatty acids that is induced during preadipocyte differentiation. Homology with Human Cd36. J Biol Chem. (1993) 268(24):17665–8. 10.1016/S0021-9258(19)49820-9 7688729

[56] Coburn CT Knapp FF Febbraio M Beets AL Silverstein RL Abumrad NA. Defective uptake and utilization of long chain fatty acids in muscle and adipose tissues of Cd36 knockout mice. J Biol Chem. (2000) 275(42):32523–9. 10.1074/jbc.M003826200 10913136

[57] Goudriaan JR Den Boer MAM Rensen PCN Febbraio M Kuipers F Romijn JA. Cd36 deficiency in mice impairs lipoprotein lipase-mediated triglyceride clearance. J Lipid Res. (2005) 46(10):2175–81. 10.1194/jlr.M500112-JLR200 16024917

[58] Vahdat-Lasemi F Farhoudi L Hosseinikhah SM Santos RD Sahebkar A. Angiopoietin-like protein inhibitors: promising agents for the treatment of familial hypercholesterolemia and atherogenic dyslipidemia. Atherosclerosis. (2025) 405:119235. 10.1016/j.atherosclerosis.2025.119235 40344904

[59] Surendran RP Visser ME Heemelaar S Wang J Peter J Defesche JC. Mutations in LPL, APOC2, APOA5, GPIHBP1, and LMF1 in patients with severe hypertriglyceridaemia. J Intern Med. (2012) 272(2):185–96. 10.1111/j.1365-2796.2012.02516.x 22239554 PMC3940136

[60] Xu M Zheng XM Jiang F Qiu WQ. MicroRNA-190b regulates lipid metabolism and insulin sensitivity by targeting IGF-1 and ADAMTS9 in non-alcoholic fatty liver disease. J Cell Biochem. (2018) 119(7):5864–74. 10.1002/jcb.26776 29575055

[61] Guha N Nevitt SP Francis M Böhning W Böhning D Sönksen PH. The effects of recombinant human insulin-like growth factor-1/insulin-like growth factor binding protein-3 administration on lipid and carbohydrate metabolism in recreational athletes. Clin Endocrinol (Oxf). (2021) 94(4):551–62. 10.1111/cen.14370 33249593

[62] Tamilarasan KP Temmel H Das SK Al Zoughbi W Schauer S Vesely PW. Skeletal muscle damage and impaired regeneration due to LPL-mediated lipotoxicity. Cell Death Dis. (2012) 3(7):e354. 10.1038/cddis.2012.91 22825472 PMC3406590

[63] Tao H Chang G Xu T Zhao H Zhang K Shen X. Feeding a high concentrate diet down-regulates expression of ACACA, LPL, and SCD and modifies milk composition in lactating goats. PLoS One. (2015) 10(6):e0130525. 10.1371/journal.pone.0130525 26086219 PMC4472775

[64] Ma N Abaker JA Wei G Chen H Shen X Chang G. A high-concentrate diet induces an inflammatory response and oxidative stress and depresses milk fat synthesis in the mammary gland of dairy cows. J Dairy Sci. (2022) 105(6):5493–505. 10.3168/jds.2021-21066 35346479

[65] Da Costa AS Pires VM Fontes CM Mestre Prates JA. Expression of genes controlling fat deposition in two genetically diverse beef cattle breeds fed high or low silage diets. BMC Vet Res. (2013) 9:118. 10.1186/1746-6148-9-118 23767408 PMC3691746

[66] Chodkowska KA Ciecierska A Majchrzak K Ostaszewski P Sadkowski T. Simultaneous miRNA and mRNA transcriptome profiling of differentiating equine satellite cells treated with gamma-oryzanol and exposed to hydrogen peroxide. Nutrients. (2018) 10(12):1871. 10.3390/nu10121871 30513813 PMC6316332

[67] Liu N Ji Y Yang Y Jia H Si X Jiang D. Impact of dietary crude protein level on hepatic lipid metabolism in weaned female piglets. Animals (Basel). (2021) 11(6):1829. 10.3390/ani11061829 34207398 PMC8235084

