# Supplementary material for: Recombinant human IGF-1 alleviates dyslipidemia induced by lactational maternal dietary restriction
Source: Front Pediatr. 2026 Jun 4;14:1829936. doi: 10.3389/fped.2026.1829936 (PMC13275451; doi:10.3389/fped.2026.1829936)
Supplement: Supplementary file 1 [file Supplementaryfile1.docx]

***Supplementary Material***

**Supplementary Table1**. Dietary Intake of Control Group rats with Different Body Weights at Different Postpartum Days

| Postpartum Days | Dietary Intake(g) | | |
| --- | --- | --- | --- |
|  | NO.1(390g)^†^ | NO.2(309g) ^†^ | NO.3(360g) ^†^ |
| 1 | 25.5 | 19.1 | 22.0 |
| 2 | 33.2 | 32.2 | 30.3 |
| 3 | 49.4 | 37.7 | 42.5 |
| 4 | 43.1 | 39.8 | 42.2 |
| 5 | 50.1 | 46.4 | 46.2 |
| 6 | 57.5 | 53.9 | 50.2 |
| 7 | 59.8 | 52.9 | 58.0 |
| 8 | 65.3 | 50.5 | 62.1 |
| 9 | 63.8 | 48.0 | 64.3 |
| 10 | 52.7 | 51.0 | 68.1 |
| 11 | 64.2 | 55.4 | 57.3 |
| 12 | 88.5 | 60.8 | 63.3 |
| 13 | 83.6 | 59.4 | 78.6 |
| 14 | 72.9 | 54.8 | 63.8 |
| 15 | 92.8 | 66.7 | 57.9 |
| 16 | 90.2 | 61.5 | 67.9 |
| 17 | 89.9 | 53.0 | 76.1 |
| 18 | 86.6 | 49.4 | 64.7 |
| 19 | 87.5 | 58.0 | 70.6 |
| 20 | 117.8 | 65.3 | 76.0 |
| 21 | 119.0 | 91.0 | 100.0 |

^†^Body weight of different rats on the day after parturition completion.


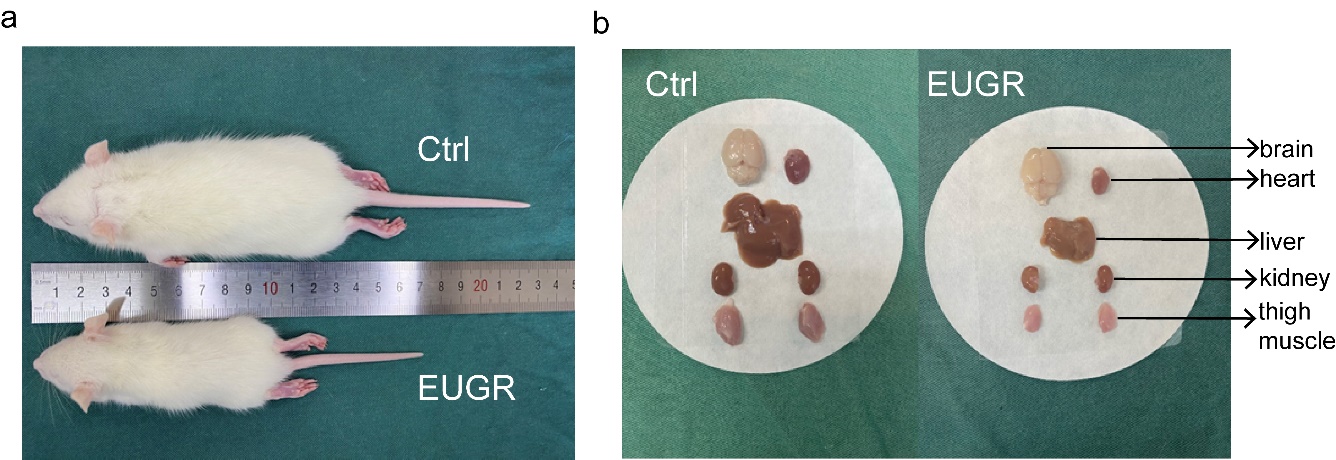


**Supplementary Figure1.** Comparative of gross body morphology (a) and visceral organs (b) in control and EUGR offspring.
